# Construction of Petal-Like Ag NWs@NiCoP with Three-Dimensional Core-Shell Structure for Overall Water Splitting

**DOI:** 10.3390/nano12071205

**Published:** 2022-04-04

**Authors:** Fan Wang, Rui Tian, Xingzhong Guo, Yang Hou, Chang Zou, Hui Yang

**Affiliations:** 1 State Key Laboratory of Silicon Materials, School of Materials Science and Engineering, Zhejiang University, Hangzhou 310027, China; 426698@zju.edu.cn (F.W.); tianr@zju.edu.cn (R.T.); 21926018@zju.edu.cn (C.Z.); yanghui@zju.edu.cn (H.Y.); 2 Hangzhou Global Scientific and Technological Innovation Center, Zhejiang University, Hangzhou 311200, China; 3 Key Laboratory of Biomass Chemical Engineering of Ministry of Education, College of Chemical and Biological Engineering, Zhejiang University, Hangzhou 310027, China; yhou@zju.edu.cn

**Keywords:** overall water splitting, core-shell structure, Ag NWs, transition metal phosphides, bifunctional electrocatalysts

## Abstract

High-efficiency, good electrical conductivity and excellent performance electrocatalysts are attracting growing attention in the field of overall water splitting. In order to achieve the desirable qualities, rational construction of the structure and chemical composition of electrocatalysts is of fundamental importance. Herein, petal-like structure Ni_0.33_Co_0.67_P shells grown on conductive silver nanowires (Ag NWs) cores as bifunctional electrocatalysts for overall water splitting were synthesized through a facile hydrothermal method and phosphorization. The resultant three-dimensional core-shell petal-like structure Ag NWs@Ni_0.33_Co_0.67_P possesses excellent catalytic activities in alkaline conditions with the overpotential of 259 mV for the oxygen evolution reaction (OER), 121 mV for the hydrogen evolution reaction (HER) and a full cell voltage of 1.64 V to reach the current density of 10 mA cm^−2^. Highly conductive Ag NWs as cores and high surface area petal-like Ni_0.33_Co_0.67_P as shells can endow outstanding catalytic performance for the bifunctional electrocatalyst. Thus, the synthetic strategy of the three-dimensional core-shell structure Ag NWs@Ni_0.33_Co_0.67_P considerably advances the practice of Ag NWs toward electrocatalysts.

## 1. Introduction

The non-renewability of traditional fossil fuels and environmental issues have forced researchers to exploit more advanced renewable energy technologies to achieve cleaner energy production and conversion [1,2,3]. In this regard, electrochemical water splitting is a very promising approach for generating high-quality hydrogen fuel [4,5]. The products of electrolyzed water are hydrogen and oxygen, and the reaction has the characteristics of safety, no pollution and easy separation of products. Hydrogen is an ideal high-efficiency, zero-emission and sustainable clean energy [6]. The water splitting reaction consists of two half-reactions: the oxygen evolution reaction (OER) and the hydrogen evolution reaction (HER), both of which require efficient electrocatalysts to improve their performance [7,8]. Previous studies have found that noble metals have excellent catalytic properties for water electrolysis, but it is difficult to put them into actual industrial production based on cost considerations [9,10]. Silver materials, as a typical noble metal material, have been extensively studied as alternative conducting electrode materials in the electrochemical field, because they are inexpensive in relative comparison with those other noble metal materials [11,12]. In particular, silver nanowires (Ag NWs) are used in water electrolysis due to their high electrical conductivity, mechanical flexibility and facile synthesis [13,14].

Transition metals represented by Ni, Co and Fe have shown high catalytic activity in some studies [15,16,17]. Transition metal phosphides that feature distinct charge structures (positive charges in metal and negative charges in phosphorus) have been studied extensively because of their good catalytic properties [18,19]. In particular, NiCo phosphides have a wide application prospect because of their environmental friendliness, low cost and high electrocatalytic activity in water electrolysis. Ji et al. designed CoP nanoframe electrocatalysts, which presented bifunctional electrocatalytic reactivities for both HER and OER [20]. Ren et al. fabricated NiP/NF catalysts with excellent electrocatalytic activities and stability [21]. Lin et al. synthesized a unique CoP/NiCoP nanotadpole heterojunction structure with special morphology such as nanotadpoles, showing good HER activity over a wide pH range and with real seawater [22]. NiCo phosphides provide more diverse redox reactions and tunable interlayer space [23]. Besides, by growing on conductive substrates, NiCo phosphides help to enhance conductivity and improve charge-transfer features.

It is well known that the properties of a catalyst are significantly determined by its size, structure and morphology [19,24]. Accurate control of the electrocatalyst structure can effectively increase the surface areas and further reinforce the catalytic active sites, thereby improving the electrocatalytic performance [25,26]. Three-dimensional nanostructures have the advantages of large specific surface area, more active sites and rapid mass transmission, while one-dimensional nanowires and nanosheets suffer from low-speed electron transfer and slow electrolyte diffusion [27]. Therefore, it is necessary to effectively combine nanowires and nanosheets to form three-dimensional nanomaterials for improving electrocatalytic performance and stability. As a supporting conductive material, Ag NWs with outstanding physicochemical properties not only improve the conductivity of catalysts but also support nanosheets. By a synergistic combination of functional Ag NWs and transition metal phosphides to obtain a three-dimensional material with a core-shell structure, the electrocatalytic performance improves effectively. However, to the best of our knowledge, there are almost no reports on the application of Ag NWs composite transition metal phosphides to water splitting.

In this work, a well-designed three-dimensional core-shell petal-like Ag NWs@NiCoP architecture is synthesized by a simple hydrothermal procedure and phosphorization process. Based on the previous research of our group, appropriate Ag NWs were selected as the electrically conductive substrates to make sure that nanosheets would grow uniformly on the Ag NWs. The facile and efficient method fabricates three-dimensional core-shell Ag NWs@NiCoP with more active sites and outstanding electrical conductivity. Three-dimensional core-shell Ag NWs@NiCoP architecture electrocatalyst achieves enhanced electrocatalytic properties as a bifunctional catalyst, which endows core-shell petal-like Ag NWs@NiCoP architecture with the catalytic ability for overall water splitting.

## 2. Materials and Methods

### 2.1. Preparation of Three-Dimensional Core-Shell Petal-Like Ag NWs@Ni_0.33_Co_0.67_P

Preparation of three-dimensional core-shell petal-like Ag NWs@Ni_0.33_Co_0.67_-OH: Ag NWs were prepared using facile methods mentioned in a previous report [28]. The as-obtained Ag NWs (2 mL, 2 wt%) were dispersed in 25 mL of water ultrasonically. Next, 0.33 mmol of Ni(NO_3_)_2_·6H_2_O, 0.67 mmol of Co(NO_3_)_2_·6H_2_O, 2 mmol of NH_4_F and 4 mmol of urea were dissolved in 25 mL of Ag NWs suspension. After stirring for 30 min, the resulting solution was treated hydrothermally at 120 °C for 6 h in a Teflon-lined stainless-steel autoclave. After the autoclave was cooled down to room temperature, the products were obtained by centrifuge, washed several times with deionized water and subsequently freeze-dried. Multiple experiments were conducted concurrently every time in order to obtain enough samples.

Phosphorization of three-dimensional core-shell petal-like Ag NWs@Ni_0.33_Co_0.67_-OH: To prepare Ag NWs@Ni_0.33_Co_0.67_P electrode, 20 mg of Ag NWs@Ni_0.33_Co_0.67_-OH and 200 mg of NaH_2_PO_2_ powders were placed separately in a porcelain crucible boat in a tube furnace at 350 °C for 2 h with a heating rate of 2 °C·min^−1^ under an Ar atmosphere. Ag NWs@Ni_0.33_Co_0.67_-OH was placed at the downstream side of the furnace. After cooling to room temperature, the three-dimensional core-shell petal-like Ag NWs@Ni_0.33_Co_0.67_P was obtained. For comparison, the molar ratios of Ni(NO_3_)_2_·6H_2_O to Co(NO_3_)_2_·6H_2_O were changed to be 1:1 and 2:1, and the as-obtained samples were named Ag NWs@Ni_0.5_Co_0.5_P and Ag NWs@Ni_0.67_Co_0.33_P. Ni_0.33_Co_0.67_P was prepared using the same method without the addition of Ag NWs.

### 2.2. Characterizations

Powder X-ray diffraction (XRD) patterns were processed using an Empyrean 200,895 X-ray diffractometer with Cu Kα radiation (PANalytical, Almelo, Netherlands). The morphology was performed using scanning electron microscopy (SEM, Hitachi SU8010 (Hitachi, Tokyo, Japan)). Transmission electron microscopy (TEM) images were obtained on a Hitachi HT-7700 (Hitachi, Tokyo, Japan) equipped with an energy dispersive X-ray (EDX) spectrometer. High-resolution transmission electron microscopy (HRTEM) was recorded on a TEM (FEI Tecnai G2 F20 (Thermo Fisher Scientific, Waltham, MA, USA)). X-ray photoelectron spectroscopy (XPS) analyses were conducted on a Thermo Scientific K-Alpha (Thermo Fisher Scientific, Waltham, MA, USA). Inductively coupled plasma-mass spectrometry (ICP-MS) was carried out on an Agilent 7700 series (Agilent Thchnologies Inc., Santa Clara, CA, USA).

### 2.3. Electrochemical Measurements

All the electrochemical measurements were performed using a CHI760E workstation (Chenhua Corp., Shanghai, China) in 1.0 M KOH (pH = 13.9). Electrodes were prepared using the same methods previously mentioned in the literature [29]. The loading of catalysts was controlled to be about 2 mg·cm^−2^. As-prepared electrodes acted as the working electrode, while a Pt sheet served as the counter electrode and an Ag/AgCl electrode worked as the reference electrode. The potentials were corrected to the reversible hydrogen electrode (RHE) by the Nernst equation (E_RHE_ = E_Ag/AgCl_ + 0.059 * pH + 0.197 V), in which E_Ag/AgCl_ is measured with the Ag/AgCl reference electrode. The main electrochemical tests included cyclic voltammetry (CV), linear sweep voltammetry (LSV) and electrochemical impedance spectroscopy (EIS). First of all, the obtained electrocatalysts were activated with the CV tests at a scan rate of 50 mV s^−1^. After activation, the LSV was conducted at a scan rate of 2 mV s^−1^, and the EIS was performed at 1.6 V (−0.15 V) versus RHE for OER (HER). Stability measurements were analyzed by 3000 cycles of CV tests and chronopotentiometry for 48 h. The electrochemical active surface area (ECSA) was estimated by CV at various scanning rates from 10 to 100 mV s^−1^ in the non-faradaic region between −0.18 and −0.12 V (versus Ag/AgCl). Note that all results were calibrated by iR-compensation according to Equation (1):

E_result_ = E_RHE_-i∙R_s_, (1)

where R_s_ was obtained from the EIS Nyquist plot. The catalytic activity of electrodes was calculated by the turnover frequency (TOF) with previously reported equations and methods [30].

## 3. Results and Discussion

### 3.1. Structure of Three-Dimensional Core-Shell Petal-Like Ag NWs@Ni_0.33_Co_0.67_P

**Figure 1:**
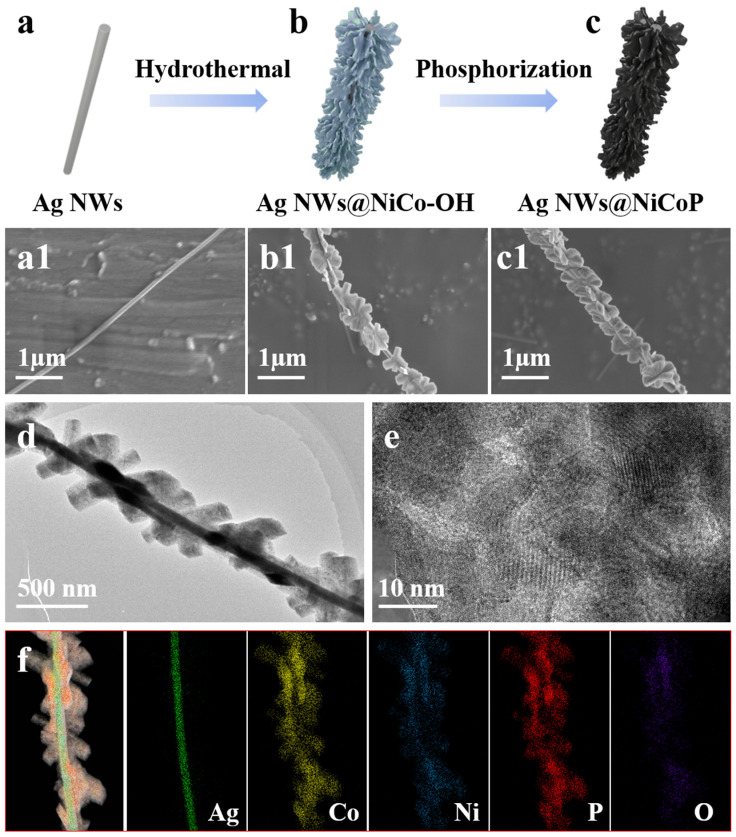
The synthesis procedures and the resulting structures for three-dimensional core-shell petal-like Ag NWs@Ni_0.33_Co_0.67_P: (**a**) Ag NWs, (**a1**) SEM image of Ag NWs, (**b**) Ag NWs@Ni_0.33_Co_0.67_-OH, (**b1**) SEM image of Ag NWs@Ni_0.33_Co_0.67_-OH, (**c**) Ag NWs@Ni_0.33_Co0_.67_P, (**c1**) SEM image of Ag NWs@Ni_0.33_Co0_.67_P, (**d**–**e**) TEM images of Ag NWs@Ni_0.33_Co0_.67_P and (**f**) elemental mapping of Ag NWs@Ni_0.33_Co_0.67_P.

The delicate processing strategy of the three-dimensional core-shell petal-like structure Ag NWs@Ni_0.33_Co_0.67_P is schematically indicated in Figure 1a–c. To fabricate the three-dimensional core-shell petal-like structure Ag NWs@Ni_0.33_Co_0.67_P, the Ag NWs@Ni_0.33_Co_0.67_-OH was firstly generated through a hydrothermal approach. The urea is decomposed in the aqueous solution to release CO_3_^2−^ and OH^−^ anions, and subsequently the Co^2+^ and Ni^2+^ cations interact with the CO_3_^2−^ and OH^−^ ions, after which plenty of nanosheets are uniformly arranged on the Ag NWs. SEM images demonstrate the diameter of smooth Ag NWs is approximately 90–120 nm (Figure 1a1 and Figure S1a), and Ni_0.33_Co_0.67_-OH nanosheets (Figure 1b1 and Figure S1b) with a diameter of around 300–400 nm are homogeneously and densely wrapped on the Ag NWs. The nanosheets are stacked and arranged in different directions to form a special three-dimensional core-shell petal-like structure [31]. During the whole heat treatment period, the entire structure can be phosphated owing to the unimpeded infiltration of phosphorus, forming an equably three-dimensional core-shell petal-like Ag NWs@Ni_0.33_Co_0.67_P structure with no collapse and aggregation (Figure 1c1 and Figure S1c). Besides, the effect of the molar ratios of Ni to Co precursor of the Ag NWs@NiCoP was investigated. From Figures S2 and S3, it can be seen that Ag NWs@Ni_0.5_Co_0.5_P and Ag NWs@Ni_0.67_Co_0.33_P still maintain the morphology of nanosheets grown on Ag NWs. With the increase of nickel content, the petal-like nanosheets disappear and gradually form hexagonal nanosheets.

Moreover, the three-dimensional core-shell petal-like structure Ag NWs@Ni_0.33_Co_0.67_P was further observed using TEM and HRTEM with the EDX spectrometer. Figure 1d depicts that the surface of Ag NWs is homogeneously covered with the petal-like Ni_0.33_Co_0.67_P nanosheets. The (111), (210) and (201) crystal planes of NiCoP phase with the lattice spacing of 0.22, 0.191 and 0.202 nm are observed in the HRTEM images, which matches well with the XRD result (Figure 1e and Figure S4). Furthermore, the corresponding EDX mapping analysis was conducted to demonstrate the distribution of the elemental components of the Ag NWs@Ni_0.33_Co_0.67_P (Figure 1f). The results indicate the existence and distinct distribution of Ag, Co, Ni, P and O throughout the nanostructure, revealing the well-defined core-shell structure with Ag NWs cores and Ni_0.33_Co_0.67_P nanosheets.

**Figure 2:**
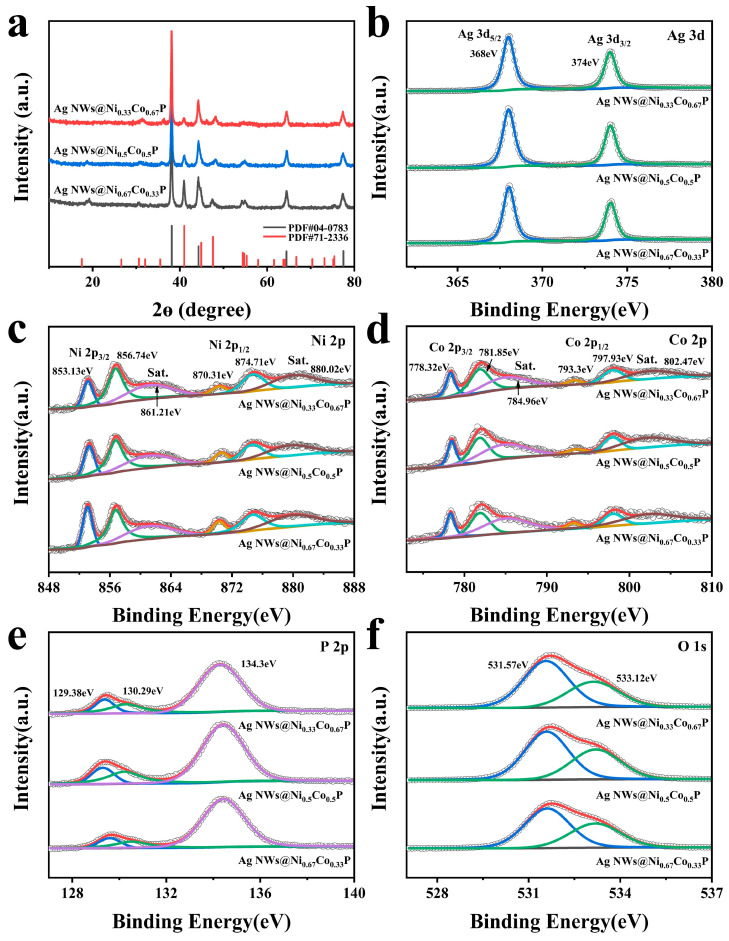
(**a**) XRD patterns of Ag NWs@NiCoP with different Ni and Co ratios. XPS spectra of Ag NWs@NiCoP with different Ni and Co ratios: (**b**) Ag 3d, (**c**) Ni 2p, (**d**) Co 2p, (**e**) P 2p, (**f**) O 1s.

XRD was conducted to elucidate the crystalline structure of the samples, as shown in Figure 2a and Figure S5. The intense peaks located at 2θ values of 38.1°, 44.2°, 64.4° and 77.4° are consistent with the Ag NWs corresponding to the (111), (200), (220) and (311) crystalline planes (PDF#04-0783) in all the curves [13]. During the hydrothermal process (Figure S5), the diffraction peaks at 19.2°, 38.6° and 52.2° can be well identified as the respective (001), (011) and (012) planes of Ni(OH)_2_ (PDF#73-1520) and the diffraction peaks located at 17.6°, 33.9°, 35.5° and 39.5° can be attributed to (020), (221), (040) and (231) planes of Co(CO_3_)_0.5_(OH)·0.11H_2_O (PDF#48-0083) [32]. It illuminates the successful fabrication of NiCo hydroxide on Ag NWs. After the phosphorization conversion, new diffraction peaks at about 40.9°, 47.5°, and 54.3° emerge (Figure 2a), which can be indexed to (111), (210), and (300) planes of NiCoP (PDF#71-2336) [19]. In addition, Table S1 confirms the atomic ratios of Ni, Co, P and Ag in the Ag NWs@NiCoP with different Ni and Co ratios.

The chemical and electronic surface states of Ag NWs@Ni_0.33_Co_0.67_P were conducted by XPS (Figure S6). In the Ag 3d spectra, Figure 2b exhibits two peaks of Ag 3d_5/2_ and Ag 3d_3/2_ at the binding energies of 368 and 374 eV in Ag NWs@Ni_0.33_Co_0.67_P, respectively, indicating the typical peak of elemental Ag [11]. In comparison with the Ag NWs@Ni_0.33_Co_0.67_-OH (Figure S7a), the peak position of Ag NWs@Ni_0.33_Co_0.67_P generates a shift. In the Ni 2p spectra (Figure S7b), the peaks located at 855.93 and 862.06 eV are assigned to the Ni(OH)_x_ and its satellite peak in the Ni 2p_3/2_ spectra for Ag NWs@Ni_0.33_Co_0.67_-OH [33]. After phosphorization, in Figure 2c, the high-resolution Ni 2p_3/2_ energy level of Ag NWs@Ni_0.33_Co_0.67_P exhibits three different chemical states with binding energies of 853.13, 856.74 and 861.21 eV associated with Ni^x+^ in NiCoP, oxidized Ni species, and the satellite of the Ni 2p_3/2_ peak, respectively [34]. Similarly, the Co 2p_3/2_ of the converted NiCoP (Figure 2d and Figure S7c) appears a new peak observed at 778.32 eV as a result of the formation of Co-P [33]. The Co 2p_3/2_ spectra in Ag NWs@Ni_0.33_Co_0.67_P exhibit main peaks at 778.32, 781.85 and 784.96 eV, which correspond to Co species in NiCoP, oxidized Co species, and the satellite of the Co 2p_3/2_ peak, respectively [35]. The other three peaks at 793.3, 797.93 and 802.47 eV accounted for the Co 2p_1/2_ core level spectra, which are indexed to the Co species in NiCoP, the oxidized Co species and the satellite of the Co 2p_1/2_ peak, respectively. In the P 2p spectra (Figure 2e), the peaks centered at 129.38 and 130.29 eV belong to the P 2p_3/2_ and P 2p_1/2_ in Ag NWs@Ni_0.33_Co_0.67_P, respectively, which are derived from NiCoP [36]. Moreover, the peak that appeared at 134.3 eV is likely to be originated from the oxidation of the samples’ surface in the environment. Figure 2f displays the O 1s binding energy for Ag NWs@Ni_0.33_Co_0.67_P to further demonstrate the oxidized Ni and oxidized Co. The peak at 531.57 eV may indicate the presence of the Ni–O and Co–O bonds [37]. Meanwhile, the peak at 533.12 eV can be illustrated as the oxidized phosphate species.

### 3.2. Electrocatalytic Performance of Three-Dimensional Core-Shell Petal-Like Ag NWs@Ni_0.33_Co_0.67_P

A standard three-electrode system was used to evaluate the electrocatalytic OER and HER performances of the as-fabricated three-dimensional core-shell petal-like Ag NWs@Ni_0.33_Co_0.67_P. Meanwhile, the performance of bare nickel foam, IrO_2_ and Pt/C were compared under identical measurement conditions.

**Figure 3:**
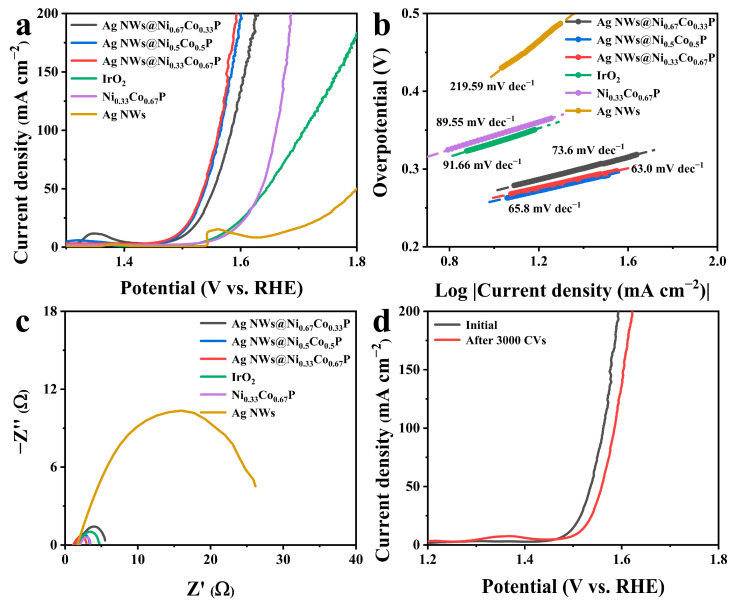
(**a**) LSV curves, (**b**) Tafel slopes and (**c**) Nyquist plots of Ag NWs@NiCoP with different Ni and Co ratios, Ni_0.33_Co_0.67_P, Ag NWs and IrO_2_ for OER; (**d**) LSV curves initially and after 3000 cycles of CV of Ag NWs@Ni_0.33_Co_0.67_P for OER.

Figure 3a shows the LSV curves for OER. The three-dimensional core-shell petal-like Ag NWs@Ni_0.33_Co_0.67_P reveals the highest OER performance, and the overpotentials are 259 and 332 mV at 10 and 100 mA·cm^−2^, respectively. The overpotential of Ag NWs@Ni_0.33_Co_0.67_P is smaller than Ag NWs, Ni_0.33_Co_0.67_P and IrO_2_. This is likely the result of the tight connection between Ag NW cores and Ni_0.33_Co_0.67_P shells that realizes rapid electron transfer and generates an intense synergistic effect. This synergistic effect exposes more active sites and effectively improves the catalytic performance. In order to demonstrate the influence of NiCoP shells grown on Ag NWs cores, Ag NWs@NiCoP with different Ni and Co ratios were carried out. Ag NWs@Ni_0.5_Co_0.5_P and Ag NWs@Ni_0.67_Co_0.33_P require overpotentials of 264 and 273 mV to drive the current density of 10 mA·cm^−2^, respectively, illustrating that Ag NWs@Ni_0.33_Co_0.67_P is an active electrocatalyst. The OER activity of bare nickel foam as catalyst substrate is measured for reference (Figure S8), illustrating that the vast majority of electrocatalytic properties of the as-prepared electrode stem from Ag NWs@Ni_0.33_Co_0.67_P catalyst. Moreover, the high OER activity of Ag NWs@Ni_0.33_Co_0.67_P is higher than other recently reported OER electrocatalysts (Table S2). The lower overpotential of Ag NWs@Ni_0.33_Co_0.67_P illustrates that the OER performance of Ag NWs@Ni_0.33_Co_0.67_P exceeds other catalysts, which results from the three-dimensional core-shell structure and phosphorization. Figure 3b reveals the smallest Tafel slope (63 mV dec^−1^) of three-dimensional core-shell petal-like Ag NWs@Ni_0.33_Co_0.67_P, which is lower than Ag NWs@Ni_0.5_Co_0.5_P (65.8 mV dec^−1^), Ag NWs@Ni_0.67_Co_0.33_P (73.6 mV dec^−1^), Ni_0.33_Co_0.67_P (89.55 mV dec^−1^), Ag NWs (219.59 mV dec^−1^) and IrO_2_ (91.66 mV dec^−1^), explaining that Ag NWs@Ni_0.33_Co_0.67_P proceeds with faster OER kinetics [38].

Furthermore, the charge transport capability of the Ag NWs@Ni_0.33_Co_0.67_P electrode was employed by electrochemical impedance spectroscopy (EIS). The semicircle diameter represents the charge-transfer resistance (R_ct_) and the horizontal intercept stands for the solution resistance (R_s_) [39,40]. The EIS results exhibit that three-dimensional core-shell petal-like Ag NWs@Ni_0.33_Co_0.67_P has the lowest R_ct_ among all the samples, which displays that the three-dimensional core-shell structure tremendously accelerates the electron transfer and has favorable reaction kinetics (Figure 3c) [41]. Simultaneously, to further clarify the advantages of phosphorization, a comparison test was conducted against the electrocatalytic performance of Ag NWs@NiCo-OH (Figure S9), which turns out that Ag NWs@NiCoP possesses more excellent catalytic activity. To estimate the OER durability of the Ag NWs@Ni_0.33_Co_0.67_P, LSV curves after 3000 cycles of CV and the chronopotentiometry test were obtained from Figure 3d and Figure S10a. LSV curves of Ag NWs@Ni_0.33_Co_0.67_P acquired before and after cycles present imperceptible change, and the OER potentials are sustainable with a negligible potential loss after 48 h, confirming excellent stability of Ag NWs@Ni_0.33_Co_0.67_P for OER electrocatalysis. However, the OER reaction is normally accompanied by the conversion of metal phosphide to metal phosphate, manifesting in the change of morphology (Figure S10b) [42]. In reality, metal phosphates play a significant role in promoting catalytic activity and stabilizing catalytic reactions [33].

**Figure 4:**
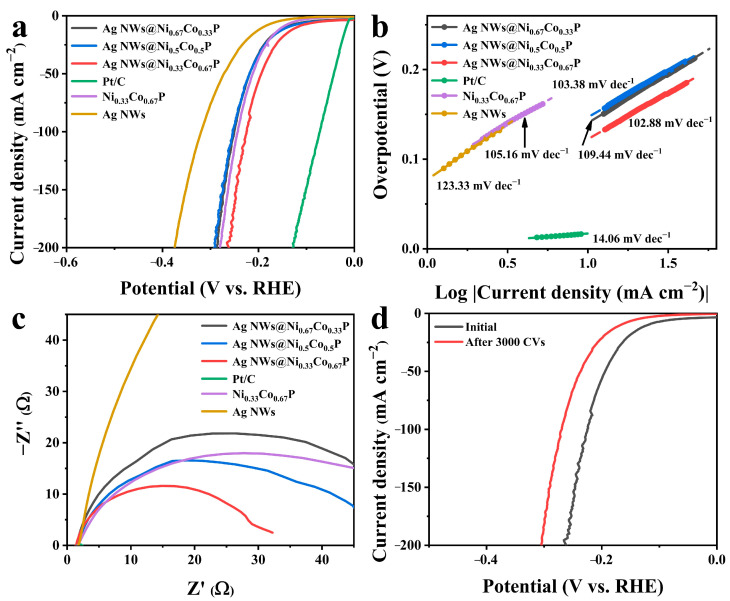
(**a**) LSV curves, (**b**) Tafel slopes and (**c**) Nyquist plots of Ag NWs@NiCoP with different Ni and Co ratios, Ni_0.33_Co_0.67_P, Ag NWs and Pt/C for HER; (**d**) LSV curves initially and after 3000 cycles of CV of Ag NWs@Ni_0.33_Co_0.67_P for HER.

Next to the OER activity, the HER performance of the three-dimensional core-shell petal-like Ag NWs@Ni_0.33_Co_0.67_P electrode was also estimated with the same conditions. Figure 4a shows the LSV curves for all the samples and Pt/C, demonstrating that three-dimensional core-shell petal-like Ag NWs@Ni_0.33_Co_0.67_P has much higher HER performance than those of Ag NWs@Ni_0.5_Co_0.5_P, Ag NWs@Ni_0.67_Co_0.33_P, Ni_0.33_Co_0.67_P and Ag NWs. Ag NWs@Ni_0.33_Co_0.67_P needs an overpotential of 121 mV to reach the current density of 10 mA cm^−2^. Furthermore, another valuable method of the Tafel plot is often used to characterize kinetics and rate control steps of the catalytic process for evaluating HER activity. The Ag NWs@Ni_0.33_Co_0.67_P displays the lowest Tafel slope of 102.88 mV·dec^−1^, compared to Ag NWs@Ni_0.5_Co_0.5_P (103.38 mV dec^−1^), Ag NWs@Ni_0.67_Co_0.33_P (109.44 mV dec^−1^), Ni_0.33_Co_0.67_P (105.16 mV dec^−1^) and Ag NWs (123.33 mV dec^−1^) (Figure 4b). The overpotential of Pt/C is 17 mV at 10 mA cm^−2^ with a Tafel slope of 14.06 mV·dec^−1^. As a blank control, the HER activity of bare nickel foam substrate was also measured (Figure S11), indicating the inert nature of HER in the range of applied potentials. In addition, the HER performance of other electrocatalysts was compared (Table S3), Ag NWs@Ni_0.33_Co_0.67_P has essentially excellent HER electrocatalytic activity.

The catalysts kinetics interface reactions of Ag NWs@Ni_0.33_Co_0.67_P was investigated by EIS in the electrocatalytic HER process. As expected (Figure 4c), the electron-transfer resistance of Ag NWs@Ni_0.33_Co_0.67_P is much lower than that of Ag NWs@Ni_0.5_Co_0.5_P and Ag NWs@Ni_0.67_Co_0.33_P, in accordance with the LSV curve and Tafel slope results. Compared with Ag NWs@NiCo-OH (Figure S12), Ag NWs@NiCoP describes much lower overpotential, smaller Tafel slope and lower R_ct_, which endows high active sites for HER. Moreover, Figure 4d and Figure S13a indicate that Ag NWs@Ni_0.33_Co_0.67_P shows an insignificant decrement in the potential after stability measurements. Besides, from the SEM images in Figure S13b, Ag NWs@Ni_0.33_Co_0.67_P after electrocatalysis retain the three-dimensional core-shell petal-like morphology, proving the robustness of the structure.

**Figure 5:**
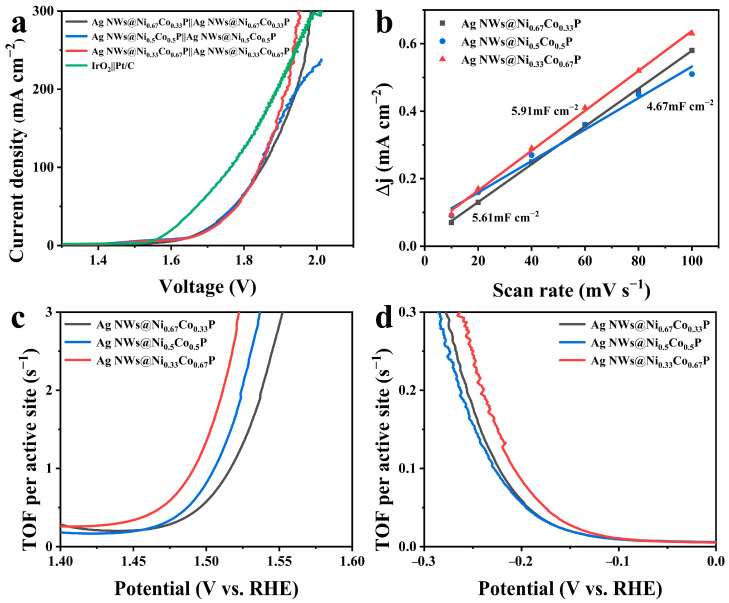
(**a**) Polarization curves of the Ag NWs@NiCoP||Ag NWs@NiCoP cell with different Ni and Co ratios and the IrO_2_||Pt/C cell for overall water splitting. (**b**) Plots of the current density versus the various scan rates for Ag NWs@NiCoP with different Ni and Co ratios; TOF curves of Ag NWs@NiCoP with different Ni and Co ratios for (**c**) OER and (**d**) HER.

Inspired by the prominent activities of the electrodes as bifunctional catalysts in alkaline solution, Ag NWs@Ni_0.33_Co_0.67_P electrodes were used as both the anode and cathode in two-electrode overall water splitting to monitor their performance. As presented in Figure 5a, Ag NWs@Ni_0.33_Co_0.67_P||Ag NWs@Ni_0.33_Co_0.67_P achieves cell voltage of 1.64 and 1.84 V to drive the current density of 10 and 100 mA cm^−2^, respectively, which are higher than those of Ag NWs@Ni_0.5_Co_0.5_P and Ag NWs@Ni_0.67_Co_0.33_P. Meanwhile, the IrO_2_||Pt/C cell voltages are 1.57 and 1.76 V, respectively. Interestingly, after the cell voltage reaches 1.93 V, the current density of the Ag NWs@Ni_0.33_Co_0.67_P||Ag NWs@Ni_0.33_Co_0.67_P exceeded that of the IrO_2_||Pt/C. Besides, the Ag NWs@Ni_0.33_Co_0.67_P electrode used for overall water splitting is outperforming other reported bifunctional electrocatalysts (Table S4). More importantly, Figure S14 declares the voltage of Ag NWs@Ni_0.33_Co_0.67_P possesses a small increase over 48 h durability measurement at a current density of 10 mA·cm^−2^, proving good stability in the long-term electrocatalytic procedure.

The XPS was performed to illustrate the changes of Ag NWs@Ni_0.33_Co_0.67_P after stability tests (Figure S15). Compared with the HER stability test, the intense anodic oxidation in the OER stability test leads to a distinct and irreversible phase transformation of metal phosphides to metal oxides/oxyhydroxides with the morphology change of Ag NWs@Ni_0.33_Co_0.67_P [38]. In reality, the useful active sites of metal phosphides for OER were confirmed to be the metal oxides/oxyhydroxides formed during the OER operation [33]. Obviously, after OER stability measurement, the lower binding energy Ni (853.13 eV) and Co (778.32 eV) peaks disappear, which are attributed to Ni-P and Co-P in the as-fabricated Ag NWs@Ni_0.33_Co_0.67_P. This result explains the phase transformation of metal phosphides to metal oxides/oxyhydroxides, which enables the supply of more active sites to facilitate catalytic reaction [43]. From Figure S15c, the lower binding energy peaks disappear in the P 2p spectra after the OER stability test, suggesting that the metal phosphides have been oxidized to metal oxides [44]. This phase transformation can be further demonstrated by the appearance of a new peak after the OER stability test (Figure S15d). The new peak at 529.4 eV in the O 1s spectra corresponds well to the M-O species, which is testified as representative electrocatalytic phases for reactivities [38]. Overall, the in situ formed metal oxides/oxyhydroxides during the OER process can be regarded as the electrocatalytically active phase.

The double-layer capacitance (C_dl_) estimates the relative ECSA via a CV test with various scan rates. Large ECSA with more exposed active sites can achieve better electrocatalytic activity [20]. Figure S16 depicts the CV curves of Ag NWs@NiCoP at different scan rates. Apparently, calculating the slope values of the current density versus the scan speed shows that Ag NWs@Ni_0.33_Co_0.67_P obtains the biggest C_dl_. The increased order of C_dl_ is Ag NWs@Ni_0.5_Co_0.5_P (4.67 mF·cm^−2^) < Ag NWs@Ni_0.67_Co_0.33_P (5.61 mF·cm^−2^) < Ag NWs@Ni_0.33_Co_0.67_P (5.91 mF·cm^−2^), illustrating that the specific surface area is availably enhanced by reasonably designing the three-dimensional core-shell structure. Meanwhile, the TOF of the catalysts evaluates the intrinsic catalytic activity of OER and HER processes [45]. Figure S17 reveals the number of active sites was calculated by CV tests with a scan rate of 50 mV·s^−1^ in 1 M phosphate buffer solution (pH = 7). Figure 5c describes the TOF value of Ag NWs@Ni_0.33_Co_0.67_P as 3.85 s^−1^ at 1.53 V for OER, which is higher than that of Ag NWs@Ni_0.5_Co_0.5_P (2.47 s^−1^) and Ag NWs@Ni_0.67_Co_0.33_P (1.54 s^−1^). Furthermore, for HER (Figure 5d), the TOF value of Ag NWs@Ni_0.33_Co_0.67_P at -0.2 V is 0.086 s^−1^, whereas the TOFs of Ag NWs@Ni_0.5_Co_0.5_P and Ag NWs@Ni_0.67_Co_0.33_P are 0.056 and 0.060 s^−1^, respectively. The biggest TOF value of Ag NWs@Ni_0.33_Co_0.67_P for OER and HER processes availably and distinctly confirmed the good catalytic activity.

The outstanding electrocatalytic performances of three-dimensional core-shell petal-like structure Ag NWs@Ni_0.33_Co_0.67_P can be assigned to the following factors: (1) three-dimensional core-shell structure generates a larger specific surface area and abundant active sites, leading to promoted electron transport and open channels for the effective release of gas; (2) the metallic nature of Ag NWs and Ni_0.33_Co_0.67_P enhance the electrical conductivity thus favors fast electron transport; (3) the intense synergistic effect between the three-dimensional core-shell structure of Ni_0.33_Co_0.67_P nanosheets and Ag NWs exposes more active sites; (4) three-dimensional core-shell structure availably reduce the electron transport resistance between the Ni_0.33_Co_0.67_P shells and Ag NWs cores, thus improving the charge-transfer ability and catalytic activity.

## 4. Conclusions

In summary, a three-dimensional core-shell petal-like structure Ag NWs@NiCoP as a bifunctional electrocatalyst was developed for overall water electrolysis. The excellent electrocatalytic performance of Ag NWs@NiCoP is attributed to its advantageous structural and chemical compositional design that results in strengthened electrical conductivity and facile electron transport. A three-dimensional core-shell structure constructed by one-dimensional Ag NWs and NiCoP nanosheets has the advantages of quick-speed electron transmission and fast diffusion of electrolytes. The as-constructed three-dimensional core-shell petal-like structure Ag NWs@NiCoP exhibits favorable catalytic performance for OER and HER. The use of Ag NWs@NiCoP as a bifunctional electrocatalyst will markedly simplify electrode preparation and facilitate its extensive application. This method provides a novel strategy for the synthesis of three-dimensional core-shell electrocatalysts by the combination of transition metal phosphide and Ag NWs.

## Data Availability

The data presented in this study are available in this article.

## Supplementary Materials

The following supporting information can be downloaded at: https://www.mdpi.com/article/10.3390/nano12071205/s1, Figure S1: SEM images of (a) Ag NWs, (b) Ag NWs@Ni_0.33_Co_0.67_-OH and (c) Ag NWs@Ni_0.33_Co_0.67_P.; Figure S2: SEM images of (a, b) Ag NWs@Ni_0.5_Co_0.5_-OH and (c, d) Ag NWs@Ni_0.5_Co_0.5_P; Figure S3: SEM images of (a, b) Ag NWs@Ni_0.67_Co_0.33_-OH and (c, d) Ag NWs@Ni_0.67_Co_0.33_P; Figure S4: HRTEM images of Ag NWs@Ni_0.33_Co_0.67_P: (a) (111), (b) (210), (c) (201); Figure S5: XRD patterns of Ag NWs@NiCo-OH with different Ni and Co ratios; Figure S6: Overall X-ray photoelectron spectroscopy (XPS) spectra of (a) Ag NWs@NiCoP and (b) Ag NWs@NiCo-OH with different Ni and Co ratios; Figure S7: XPS spectra of Ag NWs@NiCo-OH with different Ni and Co ratios: (a) Ag 3d, (b) Ni 2p, (c) Co 2p, and (d) O 1s; Figure S8: (a) LSV, (b) the corresponding Tafel slope and (c) Nyquist plot of nickel foam towards OER; Figure S9: (a) LSV curves, (b) the corresponding Tafel plots and (c) Nyquist plots of Ag NWs@NiCo-OH with different Ni and Co ratios towards OER; Figure S10: (a) Chronopotentiometry test of Ag NWs@Ni_0.33_Co_0.67_P for OER and (b) SEM images of Ag NWs@Ni_0.33_Co_0.67_P after 48 h chronopotentiometry test towards OER; Figure S11: (a) LSV, (b) the corresponding Tafel slope and (c) Nyquist plot of nickel foam towards HER; Figure S12: (a) LSV curves, (b) the corresponding Tafel plots and (c) Nyquist plots of Ag NWs@NiCo-OH with different Ni and Co ratios towards HER; Figure S13: (a) Chronopotentiometry test of Ag NWs@Ni_0.33_Co_0.67_P for HER and (b) SEM images of Ag NWs@Ni_0.33_Co_0.67_P after 48 h chronopotentiometry test towards HER; Figure S14: Chronopotentiometry test of Ag NWs@Ni_0.33_Co_0.67_P for overall water splitting; Figure S15: XPS spectra of (a) Ni 2p, (b) Co 2p, (c) P 2p and (d) O 1s of Ag NWs@Ni_0.33_Co_0.67_P before test, after HER stability test and after OER stability test; Figure S16: CV curves of (a) Ag NWs@Ni_0.67_Co_0.33_P, (b) Ag NWs@Ni_0.5_Co_0.5_P, and (c) Ag NWs@Ni_0.33_Co_0.67_P at different scan rates (10, 20, 40, 60, 80 and 100 mV S^-1^) in the non-faradaic potential region of -0.18 to -0.12 V *vs*. Ag/AgCl; Figure S17: CV curves of Ag NWs@NiCoP with different Ni and Co ratios at a scan rate of 50 mV S^-1^ in 1.0 M PBS (pH = 7) for (a) OER and (b) HER; Table S1: Metal elements content detected by ICP-MS analysis.; Table S2: Comparison of the OER performances of Ag NWs@Ni_0.33_Co_0.67_P with the previously reported electrocatalysts at alkaline media; Table S3: Comparison of the HER performances of Ag NWs@Ni_0.33_Co_0.67_P with the previously reported electrocatalysts at alkaline media; Table S4: Comparison of overall water splitting performances of Ag NWs@Ni_0.33_Co_0.67_P with the previously reported electrocatalysts at alkaline media. References [46,47,48,49,50,51,52,53,54,55,56,57,58,59,60,61] are cited in the Supplementary Materials.
