# Supplementary material for: Construction of Petal-Like Ag NWs@NiCoP with Three-Dimensional Core-Shell Structure for Overall Water Splitting
Source: Nanomaterials (Basel). 2022 Apr 4;12(7):1205. doi: 10.3390/nano12071205 (PMC9000666; doi:10.3390/nano12071205)
Supplement: Supplementary file 1 [file nanomaterials-12-01205-s001.zip › nanomaterials-1660789-supplementary.pdf]

nanomaterials

Supporting Information

Construction of Petal-Like Ag NWs@NiCoP with Three-Dimensional Core-Shell Structure for Overall Water Splitting

Fan Wang [1], Rui Tian [1], Xingzhong Guo [1,2,*], Yang Hou [3], Chang Zou [1] and Hui Yang [1]

1 State Key Laboratory of Silicon Materials, School of Materials Science and Engineering, Zhejiang University, Hangzhou 310027, China; 426698@zju.edu.cn (F.W.); tianr@zju.edu.cn (R.T.); 21926018@zju.edu.cn (C.Z.); yanghui@zju.edu.cn (H.Y.)
2 Hangzhou Global Scientific and Technological Innovation Center, Zhejiang University, Hangzhou 311200, China
3 Key Laboratory of Biomass Chemical Engineering of Ministry of Education College of Chemical and Biological Engineering, Zhejiang University, Hangzhou 310027, China; yhou@zju.edu.cn
* Correspondence: msewj01@zju.edu.cn

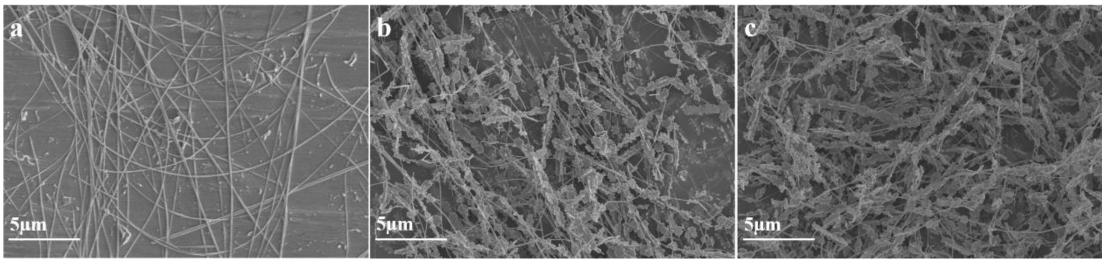

Figure S1. SEM images of (**a**) Ag NWs, (**b**) Ag NWs@$Ni_{0.33}Co_{0.67}$-OH and (**c**) Ag NWs@$Ni_{0.33}Co_{0.67}P$.

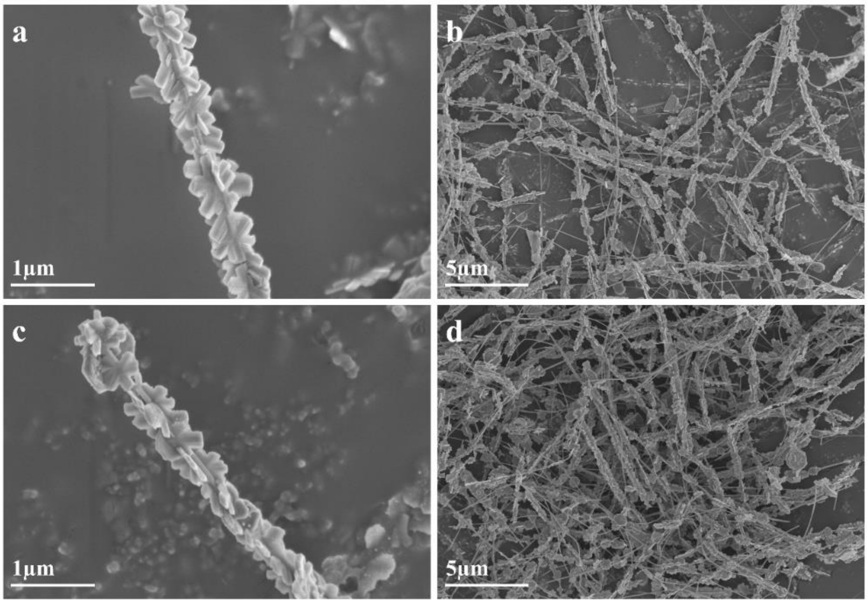

Figure S2. SEM images of (**a**, **b**) Ag NWs@$Ni_{0.5}Co_{0.5}$-OH and (**c**, **d**) Ag NWs@$Ni_{0.5}Co_{0.5}P$.

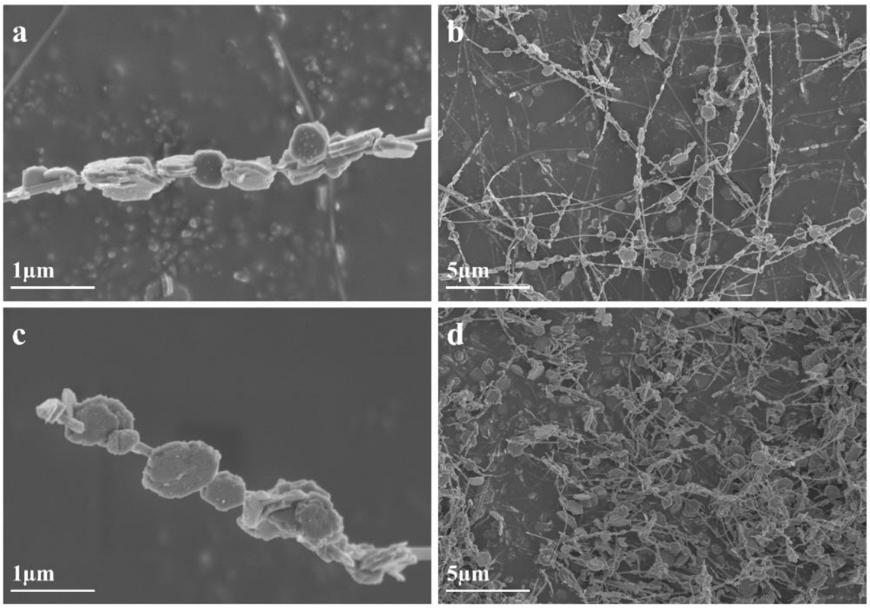

Figure S3. SEM images of (**a**, **b**) Ag NWs@$Ni_{0.67}Co_{0.33}$-OH and (**c**, **d**) Ag NWs@$Ni_{0.67}Co_{0.33}P$.

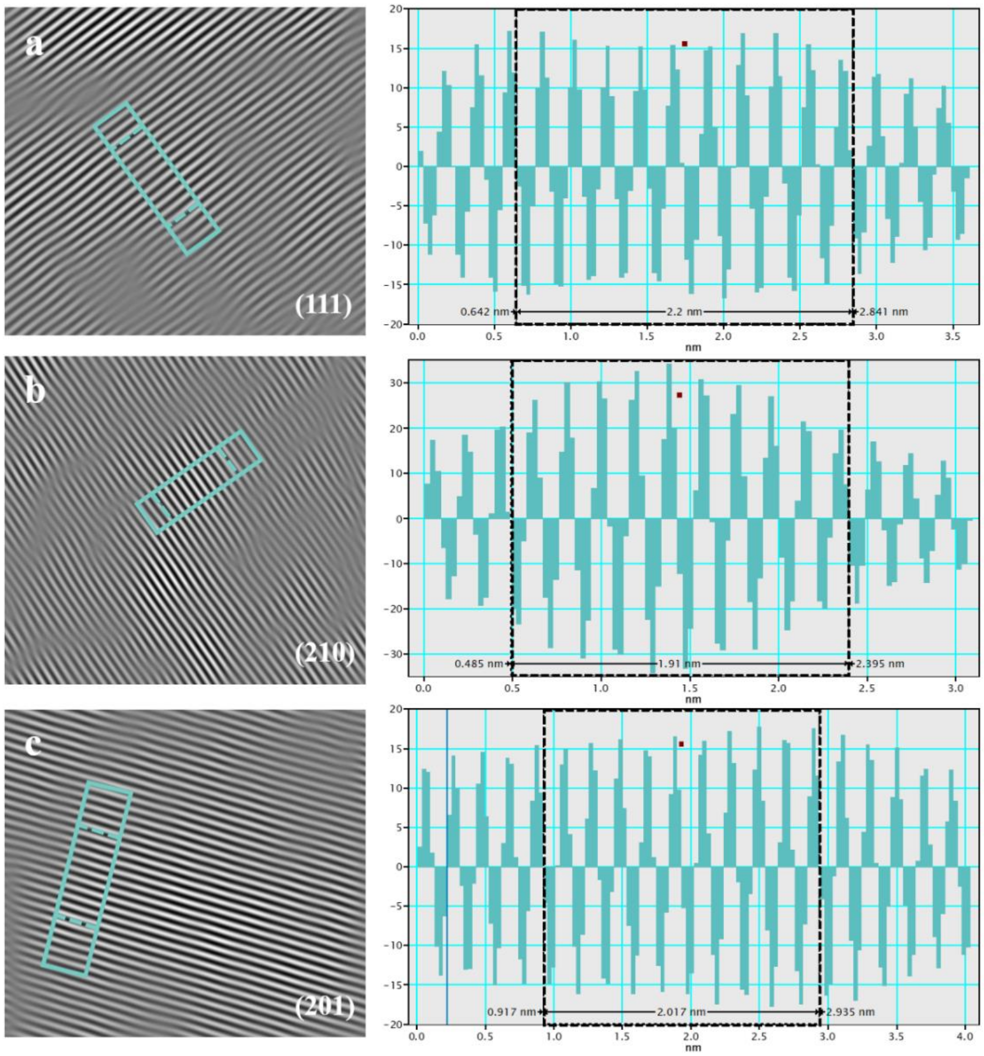

Figure S4. HRTEM images of Ag NWs@$Ni_{0.33}Co_{0.67}P$: (a) (111), (b) (210), (c) (201).

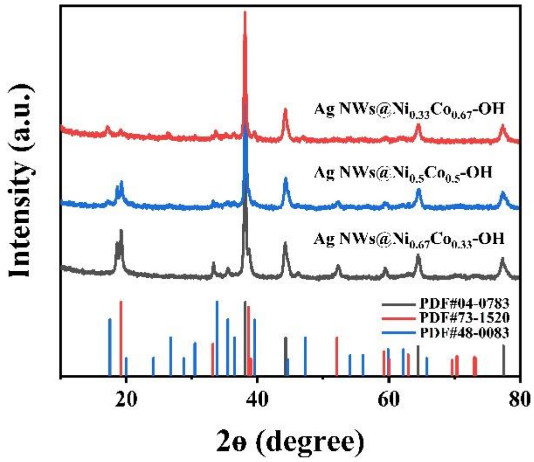

Figure S5. XRD patterns of Ag NWs@NiCo-OH with different Ni and Co ratios.

Table S1. Metal elements content detected by ICP-MS analysis.

Catalysts	Ni (at. %)	Co (at. %)	P (at. %)	Ag (at. %)
Ag NWs@$Ni_{0.33}Co_{0.67}P$	2.207	4.434	4.448	2.037
Ag NWs@$Ni_{0.5}Co_{0.5}P$	3.527	3.611	4.39	2.071
Ag NWs@$Ni_{0.67}Co_{0.33}P$	4.759	2.394	4.526	2.248

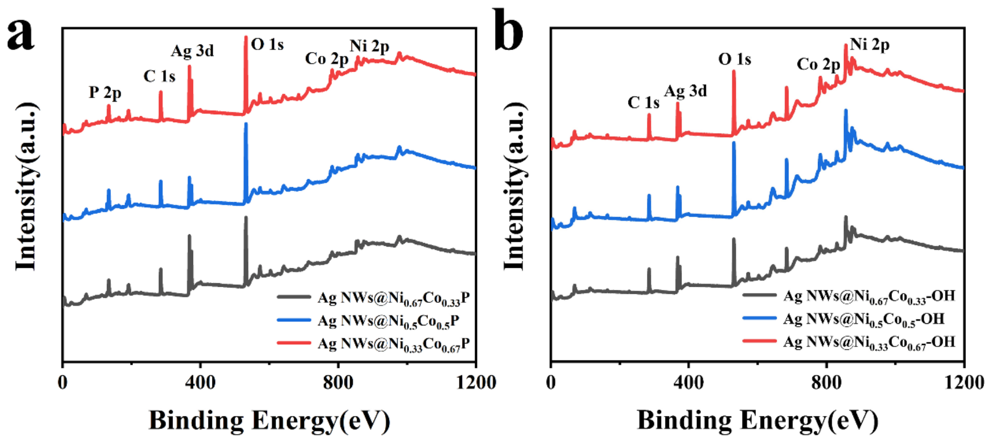

Figure S6. Overall X-ray photoelectron spectroscopy (XPS) spectra of (**a**) Ag NWs@NiCoP and (**b**) Ag NWs@NiCo-OH with different Ni and Co ratios.

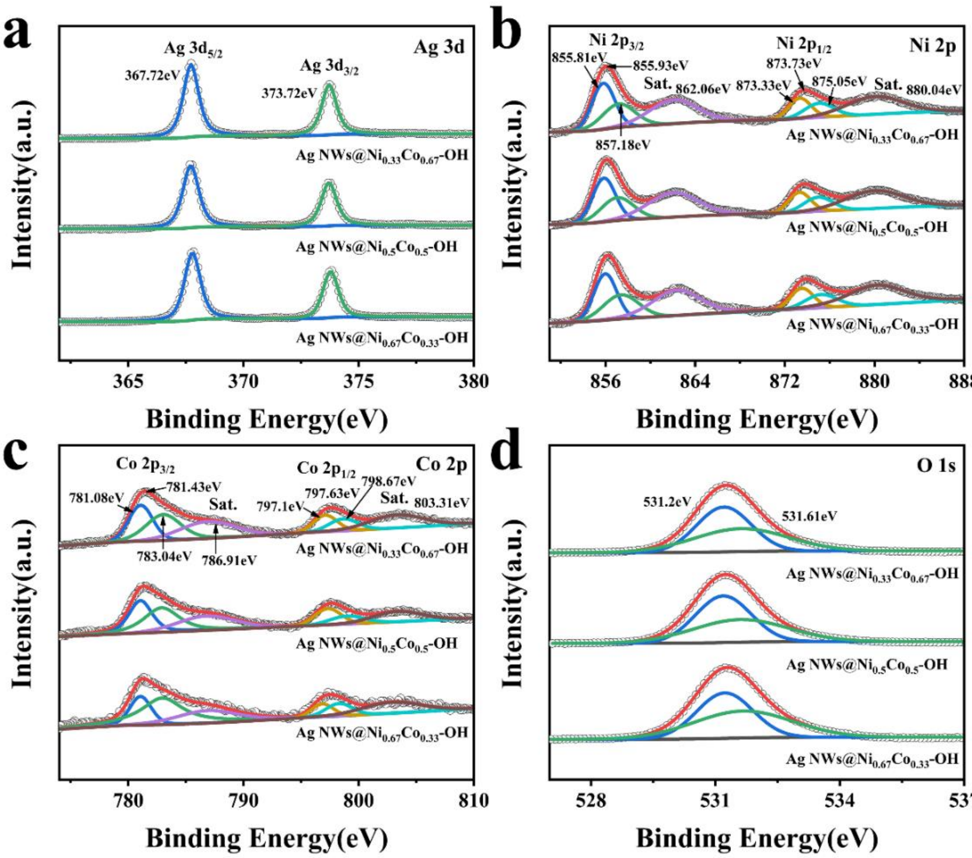

Figure S7. XPS spectra of Ag NWs@NiCo-OH with different Ni and Co ratios: (**a**) Ag 3d, (**b**) Ni 2p, (**c**) Co 2p, and (**d**) O 1s.

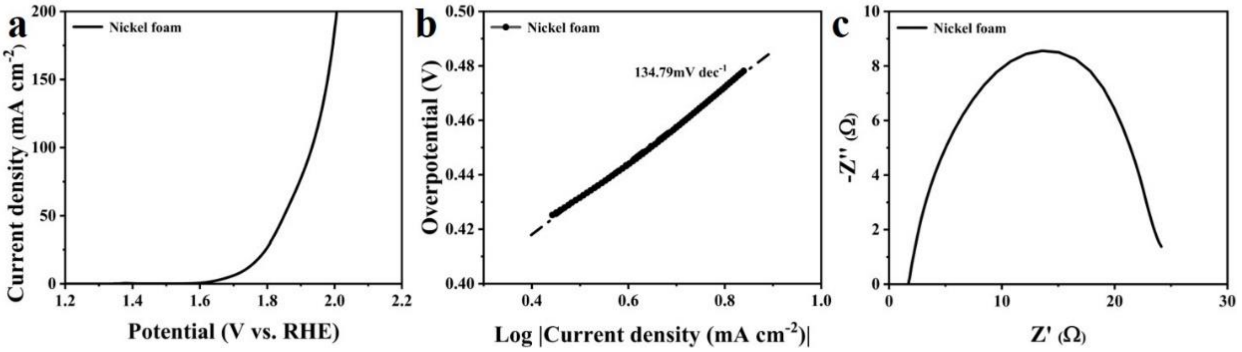

Figure S8. (**a**) LSV, (**b**) the corresponding Tafel slope and (**c**) Nyquist plot of nickel foam towards OER.

Table S2. Comparison of the OER performances of Ag NWs@$Ni_{0.33}Co_{0.67}P$ with the previously reported electrocatalysts at alkaline media.

Catalysts	Overpotential (mV) at 10 mA·cm^{-2}	Overpotential (mV) at 100 mA·cm^{-2}	Tafel slop (mV·dec^{-1})	Reference
Ag NWs@$Ni_{0.33}Co_{0.67}P$	259	332	63	This work
AgNW@NiMn-LDH(1:2)	270		40.2	[1]
Ag NWs@$Ni_{0.95}Fe_{0.05}$LDHs	330		89	[2]
Ag NWs-ZIF67	316		48.5	[3]
Ag NWs/CoO	366		146	[4]
CoP NFs	323		49.6	[5]
$NiCo_2O_4$/NiCoP	295	369	70	[6]
NiCoP/rGO	270		65.7	[7]
NiCoP/NF	280		87	[8]
$Ni_{0.6}Co_{1.4}P$ nanocages	300		80	[9]
NiCoP/C nanoboxes	330		96	[10]
hierarchical Ni-Co-P HNBs	270		76	[11]
$Ni_xCo_{3-x}O_4$ nanoneedle	320		38	[12]
NiCo-LDH/SS	270		61	[13]
(Ni,Co)S_2	270		58	[14]
NC/Co/CoP/CP	350		94	[15]
$Co_{1-x}S$@C	260		85	[16]
V-doped CoP	340		95.7	[17]
NF-CoCo-LDH	285		95	[18]
CoP NPs/CNSs	340		102.1	[19]
CoMoP@N, P-C	296		97	[20]

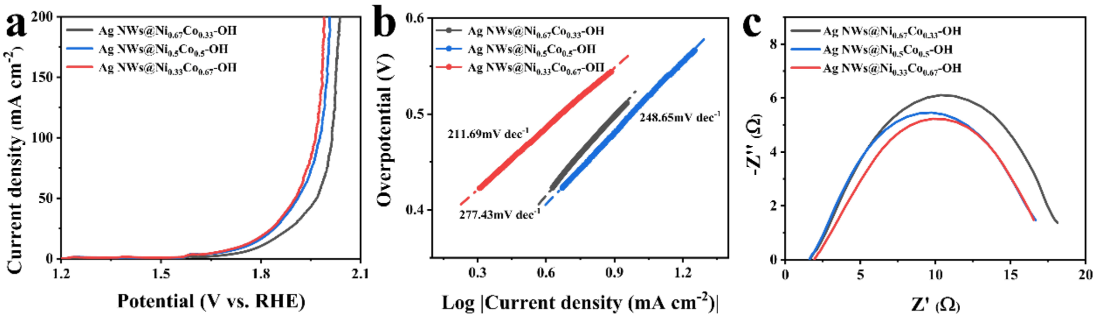

Figure S9. (**a**) LSV curves, (**b**) the corresponding Tafel plots and (**c**) Nyquist plots of Ag NWs@NiCo-OH with different Ni and Co ratios towards OER.

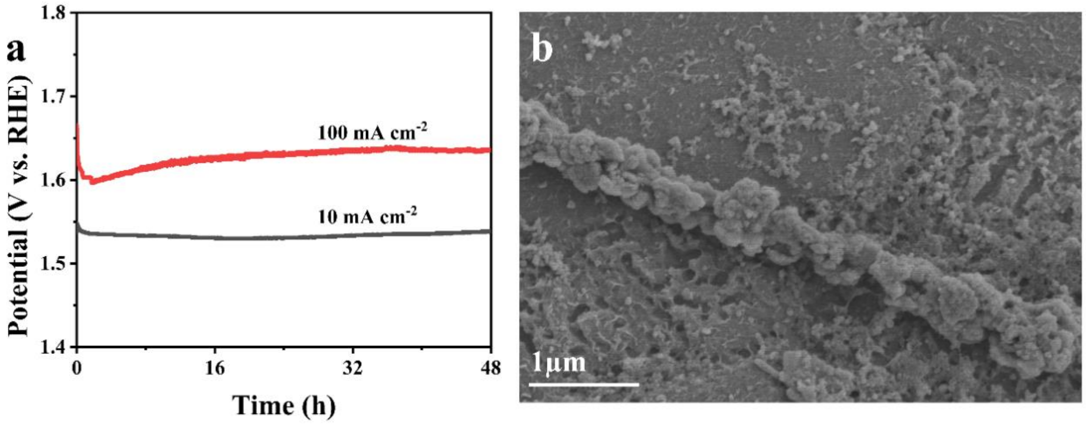

Figure S10. (**a**) Chronopotentiometry test of Ag NWs@$Ni_{0.33}Co_{0.67}P$ for OER and (**b**) SEM images of Ag NWs@$Ni_{0.33}Co_{0.67}P$ after 48 h chronopotentiometry test towards OER.

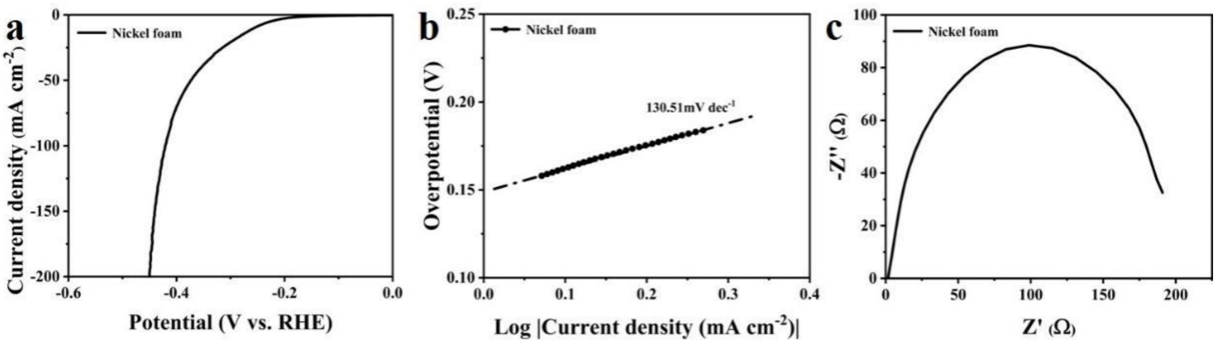

Figure S11. (**a**) LSV, (**b**) the corresponding Tafel slope and (**c**) Nyquist plot of nickel foam towards HER.

Table S3. Comparison of the HER performances of Ag NWs@$Ni_{0.33}Co_{0.67}P$ with the previously reported electrocatalysts at alkaline media.

Catalysts	Overpotential (mV) at 10 mA·cm^{-2}	Overpotential (mV) at 100 mA·cm^{-2}	Tafel slop (mV·dec^{-1})	Reference
Ag NWs@$Ni_{0.33}Co_{0.67}P$	121	225	102.88	This work
Ag NWs@$Ni(OH)_2$	123		76	[21]
Ag NWs@$Ni_{0.95}Fe_{0.05}$LDHs	200		87	[2]
Ag NWs/CoO	220		244	[4]
CoP NFs	136		56.2	[5]
$NiCo_2O_4$/NiCoP	198	303	91	[6]
NiCoP/rGO	209		124.1	[7]
CoP/NiCoP	133		88	[22]
$Ni_xCo_{3-x}O_4$ nanoneedle	170		98	[12]
NCS-1	282			[23]
Co_9S_8@NiCo LDH/NF	168		103	[24]
$(Ni,Co)S_2$	210		68	[14]
NC/Co/CoP/CP	208		126	[15]
V-doped CoP	235		91.4	[17]
CoP NPs/CNSs	115	290	90	[19]
CoNiMn/NC	191		64.38	[25]
NiFe HNSs	189		78.2	[26]
CoMoP@N, P-C	152		76.8	[20]

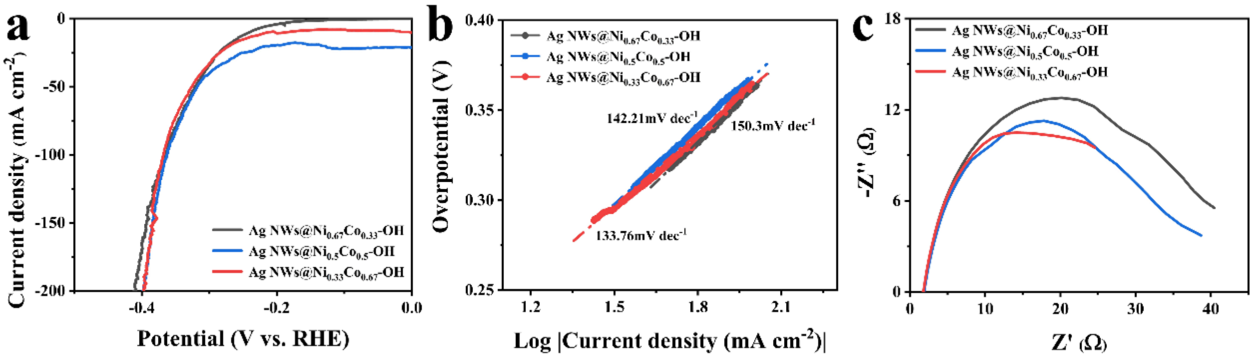

Figure S12. (**a**) LSV curves, (**b**) the corresponding Tafel plots and (**c**) Nyquist plots of Ag NWs@NiCo-OH with different Ni and Co ratios towards HER.

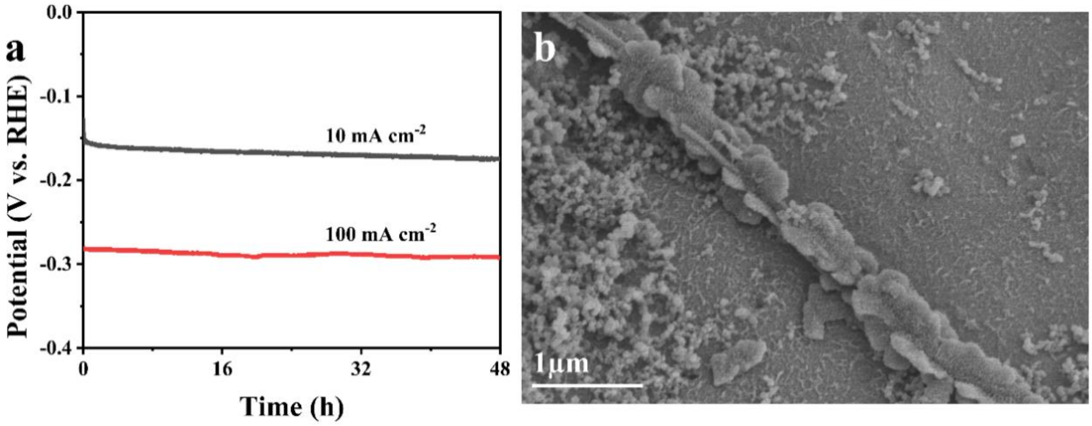

Figure S13. (**a**) Chronopotentiometry test of Ag NWs@$Ni_{0.33}Co_{0.67}P$ for HER and (**b**) SEM images of Ag NWs@$Ni_{0.33}Co_{0.67}P$ after 48 h chronopotentiometry test towards HER.

Table S4. Comparison of overall water splitting performances of Ag NWs@$Ni_{0.33}Co_{0.67}P$ with the previously reported electrocatalysts at alkaline media.

Catalysts	Voltage (V) at 10 mA·cm^{-2}	Voltage (V) at 100 mA·cm^{-2}	Reference
Ag NWs@$Ni_{0.33}Co_{0.67}P$	1.64	1.84	This work
Ag NWs@$Ni_{0.95}Fe_{0.05}$LDHs	1.7		[2]
Ag NWs/CoO	1.9		[4]
CoP NFs	1.65		[5]
$NiCo_2O_4$/NiCoP	1.66		[6]
$(Ni,Co)S_2$	1.71		[14]
NC/Co/CoP/CP	1.72		[15]
$Ni_{0.85}Se$	1.66		[27]
NiFe HNSs	1.67		[26]

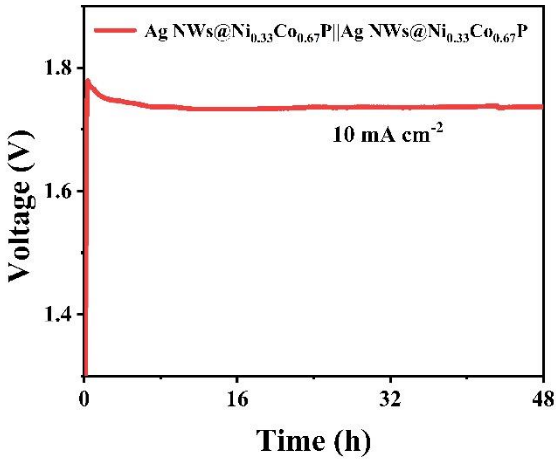

Figure S14. Chronopotentiometry test of Ag NWs@$Ni_{0.33}Co_{0.67}P$ for overall water splitting.

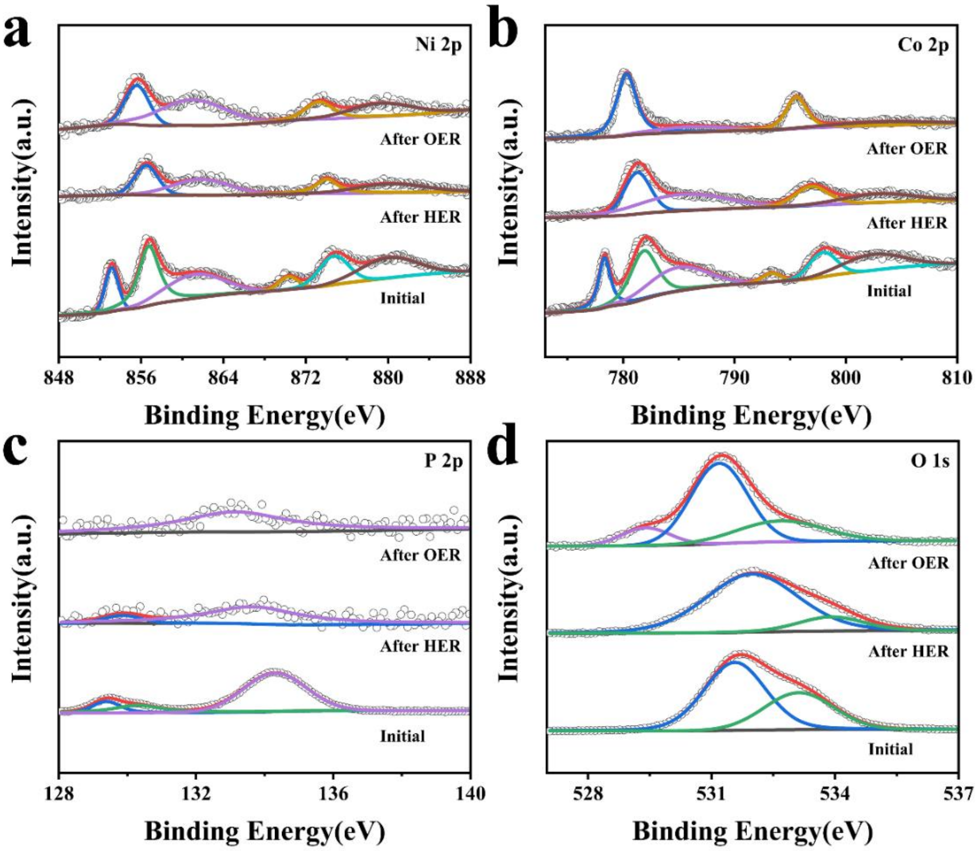

Figure S15. XPS spectra of (**a**) Ni 2p, (**b**) Co 2p, (**c**) P 2p and (**d**) O 1s of Ag NWs@$Ni_{0.33}Co_{0.67}P$ before test, after HER stability test and after OER stability test.

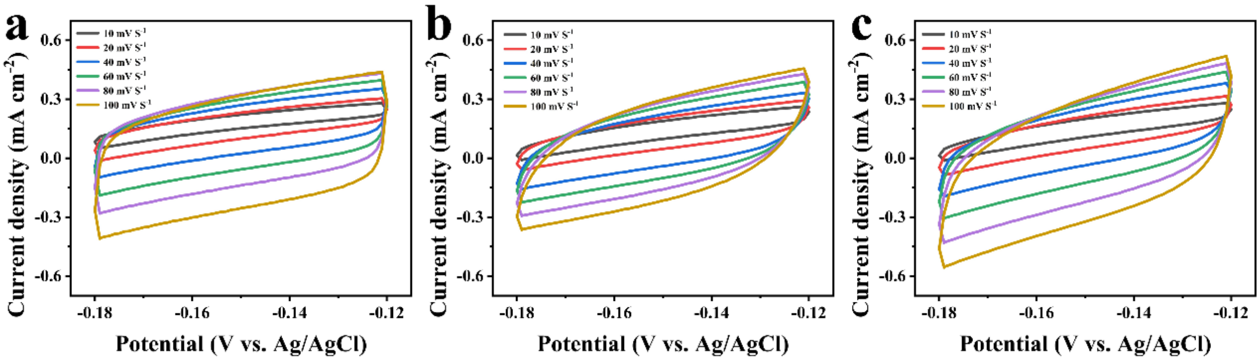

Figure S16. CV curves of (**a**) Ag NWs@$Ni_{0.67}Co_{0.33}P$, (**b**) Ag NWs@$Ni_{0.5}Co_{0.5}P$, and (**c**) Ag NWs@$Ni_{0.33}Co_{0.67}P$ at different scan rates (10, 20, 40, 60, 80 and 100 mV S^{-1}) in the non-faradaic potential region of -0.18 to -0.12 V *vs*. Ag/AgCl.

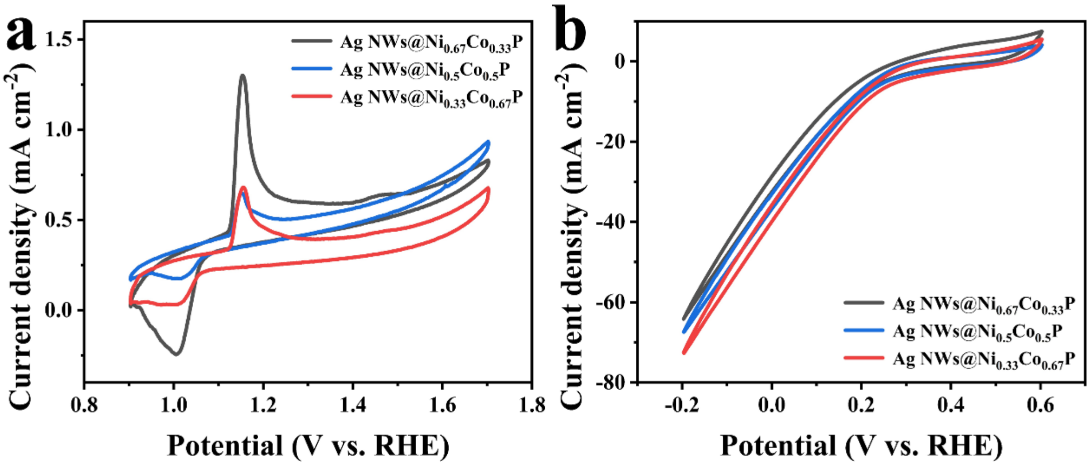

Figure S17. CV curves of Ag NWs@NiCoP with different Ni and Co ratios at a scan rate of 50 mV S^{-1} in 1.0 M PBS (pH=7) for (**a**) OER and (**b**) HER.

References

1. Chala, S.A.; Tsai, M.C.; Su, W.N.; Ibrahim, K.B.; Thirumalraj, B.; Chan, T.S.; Lee, J.F.; Dai, H.; Hwang, B.J. Hierarchical 3D architectured Ag nanowires shelled with NiMn-layered double hydroxide as an efficient bifunctional oxygen electrocatalyst. *ACS Nano* **2020**, 14, 1770–1782.
2. Zhang, X.; Marianov, A.N.; Jiang, Y.; Cazorla, C.; Chu, D. Hierarchically constructed silver nanowire@nickel-iron layered double hydroxide nanostructures for electrocatalytic water splitting. *ACS Appl. Nano Mater*. **2019**, *3*, 887–895.
3. Zhao, M.; Wang, Y.; Zhao, Q.; Li, Q.; Pang, H. Facile synthesis of silver nanowire-zeolitic imidazolate framework 67 composites as high-performance bifunctional oxygen catalysts. *Nanoscale* **2018**, *10*, 15755–15762.
4. Liu, X.; Wang, R.; He, Y.; Ni, Z.; Su, N.; Guo, R.; Zhao, Y.; You, J.; Yi, T. Construction of alternating layered quasi-three-dimensional electrode Ag NWs/CoO for water splitting: A discussion of catalytic mechanism. *Electrochim. Acta* **2019**, *317*, 468–477.
5. Ji, L.; Wang, J.; Teng, X.; Meyer, T.J.; Chen, Z. CoP Nanoframes as Bifunctional Electrocatalysts for Efficient Overall Water Splitting. *ACS Catal.* **2019**, *10*, 412–419.
6. Jin, W.; Chen, J.; Wu, H.; Zang, N.; Li, Q.; Cai, W.; Wu, Z. Interface engineering of oxygen-vacancy-rich $NiCo_2O_4$/NiCoP heterostructure as an efficient bifunctional electrocatalyst for overall water splitting. *Catal. Sci. Technol.* **2020**, *10*, 5559–5565.
7. Li, J.; Yan, M.; Zhou, X.; Huang, Z.-Q.; Xia, Z.; Chang, C.-R.; Ma, Y.; Qu, Y. Mechanistic Insights on Ternary $Ni_{2-x}Co_xP$ for Hydrogen Evolution and Their Hybrids with Graphene as Highly Efficient and Robust Catalysts for Overall Water Splitting. *Adv. Funct. Mater.* **2016**, *26*, 6785–6796.
8. Liang, H.; Gandi, A.N.; Anjum, D.H.; Wang, X.; Schwingenschlogl, U.; Alshareef, H.N. Plasma-Assisted Synthesis of NiCoP for Efficient Overall Water Splitting. *Nano Lett.* **2016**, *16*, 7718–7725.
9. Qiu, B.; Cai, L.; Wang, Y.; Lin, Z.; Zuo, Y.; Wang, M.; Chai, Y. Fabrication of Nickel-Cobalt Bimetal Phosphide Nanocages for Enhanced Oxygen Evolution Catalysis. *Adv. Funct. Mater.* **2018**, *28*, 1706008.
10. He, P.; Yu, X.Y.; Lou, X.W. Carbon-Incorporated Nickel-Cobalt Mixed Metal Phosphide Nanoboxes with Enhanced Electrocatalytic Activity for Oxygen Evolution. *Angew. Chem. Int. Ed. Engl.* **2017**, *56*, 3897–3900.
11. Hu, E.; Feng, Y.; Nai, J.; Zhao, D.; Hu, Y.; Lou, X.W. Construction of hierarchical Ni-Co-P hollow nanobricks with oriented nanosheets for efficient overall water splitting. *Energy Environ. Sci.* **2018**, *11*, 872–880.
12. Bao, J.; Liu, W.; Xie, J.; Xu, L.; Guan, M.; Lei, F.; Zhao, Y.; Huang, Y.; Xia, J.; Li, H. $Ni_xCo_{3-x}O_4$ nanoneedle arrays grown on Ni foam as an efficient bifunctional electrocatalyst for full water splitting. *Chem. Asian J.* **2019**, *14*, 480–485.
13. Jadhav, H.S.; Lim, A.C.; Roy, A.; Seo, J.G. Room-Temperature Ultrafast Synthesis of NiCo-Layered Double Hydroxide as an Excellent Electrocatalyst for Water Oxidation. *Chem.* **2019**, *4*, 2409–2415.
14. Zhang, J.; Bai, X.; Wang, T.; Xiao, W.; Xi, P.; Wang, J.; Gao, D.; Wang, J. Bimetallic Nickel Cobalt Sulfide as Efficient Electrocatalyst for Zn-Air Battery and Water Splitting. *Nanomicro Lett.* **2019**, *11*, 1-13.
15. Cong, M.; Sun, D.; Zhang, L.; Ding, X. In situ assembly of metal-organic framework-derived N-doped carbon/Co/CoP catalysts on carbon paper for water splitting in alkaline electrolytes. *Chin. J. Catal.* **2020**, *41*, 242–248.
16. He, D.; Wu, X.; Liu, W.; Lei, C.; Yu, C.; Zheng, G.; Pan, J.; Lei, L.; Zhang, X. $Co_{1-x}S$ embedded in porous carbon derived from metal organic framework as a highly efficient electrocatalyst for oxygen evolution reaction. *Chin. Chem. Lett.* **2019**, *30*, 229–233.
17. Qin, J.-F.; Lin, J.-H.; Chen, T.-S.; Liu, D.-P.; Xie, J.-Y.; Guo, B.-Y.; Wang, L.; Chai, Y.-M.; Dong, B. Facile synthesis of V-doped CoP nanoparticles as bifunctional electrocatalyst for efficient water splitting. *J. Energy Chem.* **2019**, *39*, 182–187.

18. Tolstoy, V.P.; Lobinsky, A.A.; Kaneva, M.V. Features of inorganic nanocrystals formation in conditions of successive ionic layers deposition in water solutions and the Co(II)Co(III) 2D layered double hydroxide synthesis. *J. Mol. Liq.* **2019**, *282*, 32–38.
19. Zou, W.; Dou, K.; Jiang, Q.; Xiang, J.; Kaun, C.-C.; Tang, H. Nearly spherical CoP nanoparticle/carbon nanosheet hybrids: a high-performance trifunctional electrocatalyst for oxygen reduction and water splitting. *RSC Adv.* **2019**, *9*, 39951–39957.
20. Sun, D.; Lin, S.; Yu, Y.; Liu, S.; Meng, F.; Du, G.; Xu, B. One-pot synthesis of N and P Co-doped carbon layer stabilized cobalt-doped MoP 3D porous structure for enhanced overall water splitting. *J. Alloy. Compd.* **2022**, *895*, 162595.
21. Liu, Y.; Zhang, G.; Zuo, C.; Zhao, K.; Zeng, J.; Yin, J.; Chen, H.; Xie, S.; Qiu, Y. Core-Shell AgNWs@$Ni(OH)_2$ Nanowires Anchored on Filter Paper for Efficient Hydrogen Evolution Reaction. *J. Electrochem. Soc.* **2020**, *167*, 116520.
22. Lin, Y.; Sun, K.; Liu, S.; Chen, X.; Cheng, Y.; Cheong, W.C.; Chen, Z.; Zheng, L.; Zhang, J.; Li, X.; Pan, Y.; Chen, C. Construction of CoP/NiCoP Nanotadpoles Heterojunction Interface for Wide pH Hydrogen Evolution Electrocatalysis and Supercapacitor. *Adv. Energy Mater.* **2019**, *9*, 1901213.
23. Tie, J.; Han, J.; Diao, G.; Liu, J.; Xie, Z.; Cheng, G.; Sun, M.; Yu, L. Controllable synthesis of hierarchical nickel cobalt sulfide with enhanced electrochemical activity. *Appl. Surf. Sci.* **2018**, *435*, 187–194.
24. Yan, J.; Chen, L.; Liang, X. Co_9S_8 nanowires@NiCo LDH nanosheets arrays on nickel foams towards efficient overall water splitting. *Sci. Bull.* **2019**, *64*, 158–165.
25. Jiang, B.; Li, Z. MOF-derived Co, Ni, Mn co-doped N-enriched hollow carbon for efficient hydrogen evolution reaction catalysis. *J. Solid State Chem.* **2021**, *295*, 121912.
26. Sun, X.; Shao, Q.; Pi, Y.; Guo, J.; Huang, X. A general approach to synthesise ultrathin NiM (M = Fe, Co, Mn) hydroxide nanosheets as high-performance low-cost electrocatalysts for overall water splitting. *J. Mater. Chem. A* **2017**, *5*, 7769–7775.
27. Yang, C.; Zhang, J.; Gao, G.; Liu, D.; Liu, R.; Fan, R.; Gan, S.; Wang, Y.; Wang, Y. 3D metallic Ti@$Ni_{0.85}Se$ with triple hierarchy as high-efficiency electrocatalyst for overall water splitting. *ChemSusChem* **2019**, *12*, 2271–2277.
